# Impaired conditioned pain modulation was restored after a single exercise session in individuals with and without fibromyalgia

**DOI:** 10.1097/PR9.0000000000000996

**Published:** 2022-04-01

**Authors:** Ali Alsouhibani, Marie Hoeger Bement

**Affiliations:** a Clinical & Translational Rehabilitation Health Science Program, Department of Physical Therapy, College of Health Sciences, Marquette University, Milwaukee, WI, USA; b Department of Physical Therapy, College of Medical Rehabilitation, Qassim University, Buraydah, Saudi Arabia

**Keywords:** Pain, Exercise, Conditioned pain modulation, Fibromyalgia, Body composition, Physical activity

## Abstract

**Introduction:**

Exercise is an effective nonpharmacological intervention for individuals with fibromyalgia syndrome (FMS); however, considerable variability is observed in their pain response after a single exercise session that could be due to differences in baseline central pain inhibition (ie, conditioned pain modulation [CPM]).

**Objectives:**

This study examined the effect of isometric exercise on CPM in people with FMS and control participants. A subaim was to identify whether pain inhibition after exercise was due to differences in baseline CPM.

**Methods:**

Twenty-one individuals with FMS (50.5 ± 14.9 years) and 22 age-matched and sex-matched controls (49.2 ± 13.3 years) participated in a familiarization session and 2 randomized experimental sessions: (1) low-intensity isometric exercise and (2) quiet rest control. Conditioned pain modulation was measured before and after each experimental session. In addition, body composition and physical activity levels were collected to determine potential group differences.

**Results:**

Both groups had comparable body composition and physical activity levels and reported similar exercise-induced hypoalgesia (increase in pressure pain thresholds) at the exercising muscle (quadriceps muscle) and systemically (deltoid muscle). Both groups had a decrease in CPM after exercise and quiet rest; however, in both FMS and control participants with impaired baseline CPM, there was an increase in CPM at the deltoid muscle after exercise.

**Conclusion:**

In persons with low CPM, irrespective of health status, isometric exercise enhanced CPM at a site distal from the exercising muscle. Our results support the use of isometric exercise when initiating an exercise program especially for individuals with impaired CPM.

## Abstract 2

Supplemental Digital Content is Available in the Text.

Submaximal isometric exercise improved impaired conditioned pain modulation acutely in individuals with and without fibromyalgia, regardless of health status.

## 1. Introduction

People with fibromyalgia syndrome (FMS) report chronic widespread pain and demonstrate abnormal endogenous pain modulation including reduced conditioned pain modulation (CPM) and exercise-induced hypoalgesia (EIH).^23^ Exercise-induced hypoalgesia is a phenomenon in which pain decreases with exercise^17^ and may occur locally at the exercising muscle^21,24,39^ or systemically^16,22,24,26^ at remote sites. For the management of FMS, exercise is considered a first-line treatment. Despite the benefits of long-term exercise training, people with FMS report inconsistent pain relief after a single exercise session^18^ in addition to inconsistent CPM responses.^2^

Previous research has shown that CPM predicts EIH in young and older healthy adults^27,38^ and in people with chronic musculoskeletal pain.^40^ In addition, we have shown that individuals who exhibit systemic EIH after an acute bout of isometric exercise have a significantly reduced CPM response.^1^ Similarly, systemic EIH is reduced when measured immediately after CPM,^11^ suggesting that systemic EIH and CPM may have similar mechanisms. In our previous study, however, participants had a strong baseline CPM response. Therefore, it is unknown how isometric exercise affects CPM in individuals with an impaired CPM response.

People may respond differently to treatments based on whether they have a functioning CPM response (ie, CPM responder) compared with an abnormal CPM response (ie, CPM nonresponder).^3,5,34,46^ For example, treatments such as joint mobilization and transcutaneous electrical nerve stimulation (TENS) have been shown to restore CPM.^3,5^ Relevant to exercise, transcranial direct current stimulation to the motor cortex has been shown to enhance the CPM response.^9^ Because exercise activates the motor cortex, it may potentially restore CPM in individuals with inefficient CPM. Understanding the effects of a single exercise session on central pain inhibition may provide insight to improving exercise tolerance and transition to regular exercise participation. Furthermore, isometric exercise offers a great tool in nonpharmacological pain management because it can be performed anywhere, involves inexpensive equipment, and can be used in various exercise programs (eg, yoga and tai chi) by people of all fitness levels.

The aim of this study was to examine the effect of isometric exercise on pain inhibition (CPM) in people with FMS and control participants. A subaim was to identify whether pain inhibition after exercise was due to differences in baseline CPM. We hypothesized that individuals with FMS would have reduced baseline CPM compared with controls, and exercise would restore CPM in those with attenuated CPM. Factors that may affect CPM or the pain response after exercise such as body composition and physical activity levels^36^ were collected to determine potential baseline differences.

## 2. Methods

### 2.1. Participants

Before data collection, power analysis was performed with the software G*Power 3.1.9.2 to determine the sample size.^8^ For the main analyses (mixed-model analysis of variance [ANOVA]), at an α value of 0.05 and β (power) value of 0.95, our power analysis indicated that 18 participants per group would be required to detect a large effect based on previous research with a similar design.^1^ Twenty-one individuals with FMS (18 women and 3 men, mean age ± SD, 50.5 ± 14.9) and 22 age-matched and sex-matched controls (20 women and 2 men, mean age ± SD, 49.2 ± 13.3) were recruited from a large Midwestern metropolitan area (Milwaukee, WI).

Participants with FMS were diagnosed by a physician, and healthy control participants were excluded if they had acute or chronic pain. All participants were excluded if they had (1) diabetes, (2) contraindications to exercise, or (3) unstable medical or psychiatric condition. Medication use in participants with FMS was allowed if they were stable for at least 2 weeks to better mimic clinical scenarios. The Institutional Review Board at the Marquette University approved the protocol of this study, which is registered at ClinicalTrials.gov (NCT03778476).

### 2.2. Experimental design

**Figure 1.**
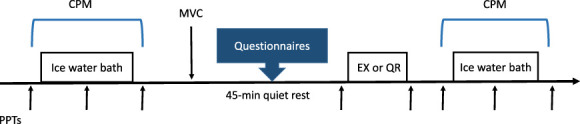
Study design. During each experimental session, CPM was measured twice (before and after exercise or quiet rest) and PPTs were measured a total of 8 times at the right quadriceps and deltoid muscles: 3 times with each of the 2 CPM protocols (before, during, and after ice water bath) and 2 times immediately before and after quiet rest or exercise (45 minutes after the first CPM trial). “↑” = PPTs at the quadriceps and deltoid muscle. CPM, conditioned pain modulation; EX, exercise; MVC, maximum voluntary contraction; PPT, pressure pain threshold; QR, quiet rest.

Participants participated in 1 familiarization session and 2 randomized experimental sessions (isometric exercise or quiet rest) with approximately 1 week separating the sessions (Fig. 1). At the beginning of the familiarization session, participants completed the written informed consent, medical history form, physical activity readiness questionnaire (PARQ), and short-form McGill questionnaire (SF-MPQ). Additional questionnaires for participants with FMS included the following: Revised Fibromyalgia Impact Questionnaire (FIQR) and 2010 American College of Rheumatology diagnostic criteria for fibromyalgia (ACR).^44^ Description of all questionnaires is included in table supplemental digital content 1 (available at http://links.lww.com/PR9/A153). Next, participants were instructed on the experimental procedures followed by a body scan (Lunar iDXA, GE Healthcare, Madison, WI) with Encore Software (version 14.10). At this time, participants were also familiarized to the pressure algometer (Somedic, Sweden), CPM (see CPM section), and exercise equipment.

During the experimental sessions (exercise or quiet rest), participants started each session by completing the SF-MPQ (all participants) and the FIQR (FMS participants only). Conditioned pain modulation was measured twice, before and after quiet rest or exercise of the right knee extensor muscles (Fig. 1). In both sessions, maximal voluntary contractions (MVC) of the right knee extensor muscles were performed immediately after the first CPM trial and used to calculate the exercise intensity (ie, 30% MVC). After completion of the MVCs, 45 minutes of rest occurred before the start of exercise or quiet rest. During this time, participants were given instructions on the Actigraph physical activity monitor in the first experimental session and completed the International Physical Activity Questionnaire (IPAQ) in the second experimental session.

### 2.3. Isometric exercise

The exercise task consisted of a submaximal isometric contraction (30% MVC) of the right knee extensor muscles held until task failure. Task failure was met when participants were unable to maintain the force within 10% of the target force for 3 of 5 consecutive seconds.^16,26,27^ This exercise task was chosen because we have shown that a low-intensity exercise held for a long duration produces the greatest hypoalgesia compared with other intensities and durations^18^ and tolerated by individuals with FMS.^19^ Positioning of participants and exercise setup has been previously described.^1^ In brief, participants were seated upright on a plinth table with their hips and knees positioned at 90° stabilized with 2 straps over their thighs (distal to the hip and proximal to the knee). A hand-held dynamometer (Commander Echo Muscle Testing Dynamometer, JTech Medical) was attached to the leg of the plinth and stabilized using Velcro straps around the leg of the participants just above the malleolus. Participants were instructed to match a target force displayed on a wireless portable monitor (Commander Echo Console, JTech Medical) during the performance of the submaximal isometric contraction and received verbal encouragement.

Participants used 0 to 10 numerical rating scales to rate their perceived exertion (RPE) and pain intensity (before the start of the exercise and every minute until the end of the exercise). Participants were allowed to stop the exercise if pain was intolerable (n = 0).

### 2.4. Pressure pain thresholds

Pressure pain thresholds (PPTs) were measured at the right quadriceps and deltoid muscles with a handheld algometer (1-cm^2^ rubber tip, delivery rate 50 kPa/s) in which participants were instructed to press a timing device when the pressure first changed to pain. Participants were familiarized to PPTs in the familiarization session by performing 3 measurements on the nail bed in addition to measurements of the quadriceps and deltoid muscles during the familiarization CPM protocol. During each experimental session, PPTs were measured 8 times: 3 with each of the 2 CPM protocols (before, during, and after ice water bath) and before and after quiet rest or exercise (Fig. 1). To minimize participants' exposure to multiple PPTs, 2 PPTs were performed at each time point with a 10-seconds interstimulus interval.^1^ The location of PPTs was shifted 1 cm up or down after the fourth PPT measurement (ie, before exercise or quiet rest) to minimize local tenderness. The 2 PPT trials recorded at each site were averaged for data analysis.

### 2.5. Conditioned pain modulation

Pressure pain thresholds were measured at the right deltoid and quadriceps muscles before, during (after 20 seconds), and after submersion of the left foot in a circulating ice water bath (6°C ± 1°C; immersion circulator, model 10000, Nomiku Inc, San Francisco, CA) for 2 minutes. Participants were instructed to keep their foot in the ice water bath for the entire 2 minutes. If a participant reportedly could not tolerate the test and removed their foot before the end of 2 minutes (n = 2), PPTs continued to be measured and included in the analyses.^1^

### 2.6. Physical activity

During the first experimental session, participants were given an activity monitor (Actigraph, wGT3X-BT, Pensacola, FL) to wear on the nondominant wrist for 7 days. Participants were encouraged to keep daily logs for sleep time, physical activity, and removal time. ActiLife software (ActiLife 6.13.1, Pensacola, FL) was used to analyze Actigraph data with “worn on wrist” correction applied. The data of 4 valid days (2 weekdays and 2 weekends) were used for all participants.^31^ Activities were divided into either sedentary or light activities or moderate-to-vigorous physical activities (MVPA) based on Freedson criteria.^10^

### 2.7. Data analysis

Data were analyzed using the IBM Statistical Package for Social Sciences (SPSS version 26, Armonk, NY) and reported as mean ± SD in the text and tables and mean ± SE in the figures. Normality was checked using the Kolmogorov–Smirnov test and visual inspection of Q–Q plots. Extreme outliers were tested with the Grubbs test and, when significant, were winsorized to one unit greater than the next outlying score.^6^ Independent *t* tests or the Mann–Whitney *U* tests for nonnormally distributed data were performed between the groups (healthy controls or FMS) to identify potential differences in characteristics. The Friedman test was used to compare whether changes occurred with SF-MPQ or FIQR across sessions.

#### 2.7.1. Baseline conditioned pain modulation

To determine whether PPTs increased at the deltoid and quadriceps muscles during or after the ice water bath (ie, CPM) in the 2 experimental sessions, a mixed-model ANOVA (session [exercise and quiet rest] × time [PPTs before, during, and after ice water bath] × site [deltoid and quadriceps]) was performed with group (healthy controls and FMS) as between-subject factor.

In addition, a mixed-model ANOVA was performed comparing CPM magnitude at baseline between sessions (quiet rest and exercise) at each site (deltoid and quadriceps) with a between-subject factor group (participants with and without FMS). All CPM analyses were performed using the relative change calculated at each site immediately after the foot was removed from ice water: ([PPT after ice water bath − PPT before ice water bath]/PPT before ice water bath) × 100; this method for CPM was chosen based on the current recommendations for measuring CPM.^45^ Conditioned pain modulation analyses using other calculation methods (ie, relative change during ice water bath and absolute change during and after ice water bath) were also performed yielding similar results; however, for simplicity, we are only reporting relative change after ice water bath.

#### 2.7.2. Pressure pain thresholds after washout period

To determine whether PPTs returned to baseline after the 45 minutes of quiet rest (ie, washout period that was performed in both experimental sessions), a mixed-model ANOVA was performed (session [exercise and quiet rest] × PPT trial [baseline PPTs and PPTs before exercise or quiet rest] × site [deltoid and quadriceps]) with group as a between-subject factor. These analyses were followed by post hoc testing with the Bonferroni correction using paired and independent *t* tests, as appropriate.

#### 2.7.3. Baseline conditioned pain modulation responders and nonresponders

Participants for both groups were categorized as CPM responders based on the change in the SEM of PPTs^41^; SEMs for each site were calculated by performing a mixed-model ANOVA for baseline PPTs of all 3 sessions and then the square root of its mean square error for both groups.^41,43^ The percent change of SEM from the average baseline PPTs were then calculated for each group at each site (eg, %SEM change = (SEM − Average PPT)/Average PPT).^29^ Participants who had a relative CPM change that was larger than the %SEM change were then classified as CPM responders from the first CPM trial of each respective session (exercise or quiet rest).^20^

#### 2.7.4. Pressure pain thresholds after exercise and quiet rest

To examine whether PPTs increased (ie, EIH) locally at the exercising muscle or systemically, mixed-model ANOVA was performed (session [exercise and quiet rest] × PPT trial [pre-exercise and postexercise or quiet rest] × site [deltoid and quadriceps]) with between-subject factor group (FMS or healthy controls). The analyses were followed by post hoc testing using paired *t* tests with the Bonferroni correction.

#### 2.7.5. Conditioned pain modulation after exercise and quiet rest

To investigate the effect of exercise on the CPM response, CPM after exercise and quiet rest were analyzed using a mixed-model ANOVA (session [exercise and quiet rest] × CPM trial [pre-exercise and postexercise or quiet rest] × site [deltoid and quadriceps]) with a between-subject factor group. To examine whether the effects of exercise on CPM are similar in responders and nonresponders, a mixed-model ANOVA (CPM trial [CPM pre-exercise and postexercise or quiet rest]) was performed for each session and each site separately with between-subject factors (group [healthy and FMS] and CPM response [responders and nonresponders]). Post hoc testing with Bonferroni-corrected paired *t* tests or the Wilcoxon signed-rank test for nonnormally distributed data were performed as appropriate.

## 3. Results

### 3.1. Participants' characteristics

Table 1 summarizes participants' characteristics. All participants completed all sessions except for 1 control participant who did not show up for the third (exercise) session. One individual with FMS refused the body scan. Five participants were excluded from accelerometery data for the following reasons: (1) refused to wear Actigraph (n = 2), (2) did not meet wear time criteria (ie, at least 4 days of wear time) (n = 2), and (3) lost Actigraph (n = 1).

**Table 1:** Participants' characteristics.

	Healthy controls	n	Fibromyalgia	n	*P*
Age (y)	49.2 ± 13.3	22	50.5 ± 14.9	21	0.763
Male		2		3	
SF-MPQ					
Total	0.82 ± 1.4	22	8.0 ± 6.5	21	**<0.001**
FIQR					
FIQR functional			26.7 ± 19.7	21	
FIQR overall impact			7.1 ± 5.2	21	
FIQR symptom			45.0 ± 20.3	21	
FIQR total			38.6 ± 19.7	21	
ACR diagnostic criteria for fibromyalgia (2010)					
ACR WPI			9.0 ± 4.0	21	
ACR symptom severity			10.1 ± 6.3	21	
Did not meet criteria				5	
Pain medications					
Acetaminophen				5	
NSAIDs				4	
Amitriptyline				2	
Tramadol				3	
Hydrocodone				1	
Gabapentin				3	
Duloxetine				2	
Milnacipran				1	
Lamotrigine				2	
Cyclobenzaprine				4	
Clonazepam				1	
Escitalopram				1	
Tizanidine				1	
Diazepam				1	
Dicyclomine				1	
Eletriptan				1	
Trazadone				1	
Exercise					
MVC (lbs.)	293.3 ± 102.0	21	289.1 ± 98.8	21	0.892
Target force (30% MVC)	88.1 ± 30.5	21	86.7 ± 29.6	21	0.878
Time to exhaustion (min)	7.0 ± 4.1	21	5.3 ± 2.3	21	0.103
Pain at the start (0–10)	0.1 ± 0.1	21	1.04 ± 0.3	21	**0.005**
RPE at the start (0–10)	0	21	0	21	
Pain at the end (0–10)	7.4 ± 2.3	21	8.5 ± 2.1	21	0.142
RPE at the end (0–10)	8.7 ± 1.5	21	9.0 ± 1.5	21	0.593
Body composition					
BMI	27.9 ± 5.7	22	31.5 ± 8.5	20	0.105
Total body fat (%)	37.3 ± 8.2	22	41.5 ± 9.0	20	0.125
Total lean mass (lbs)	100.1 ± 17.9	22	105.1 ± 17.8	20	0.376
Total body BMC (lbs)	5.6 ± 0.9	22	5.5 ± 1.1	20	0.694
Visceral fat mass (lbs)	1.6 ± 1.2	22	2.8 ± 2.0	20	**0.027**
Physical activity					
Accelerometery					
Sedentary (%)	52.8 ± 8.3	19	53.3 ± 7.3	19	0.850
Light (%)	34.0 ± 7.1	19	33.7 ± 5.9	19	0.895
Moderate or vigorous (%)	13.1 ± 5.9	19	12.9 ± 5.8	19	0.916
Self-report					
IPAQ total walking MET (min/wk)	1598.7 ± 1871.6	21	2354.8 ± 2708.7	21	0.489
IPAQ total moderate MET (min/wk)	1550.5 ± 1645.2	21	2384.2 ± 3226.1	21	0.724
IPAQ total vigorous MET (min/wk)	607.6 ± 1078.4	21	2796.2 ± 5087.2	21	0.226

ACR, American College of Rheumatology; BMI, body mass index; FIQR, Revised Fibromyalgia Impact Questionnaire; IPAQ, International Physical Activity Questionnaire; MVC, maximal voluntary contraction; RPE, rate of perceived exertion; SF-MPQ, short form McGill pain questionnaire; WPI, widespread pain index. (***P* < 0.05**)

One outlier was detected and adjusted from each of the following variables: CPM at the quadriceps before quiet rest and IPAQ total MET. Two outliers were detected and adjusted from the following variables: IPAQ total moderate MET and IPAQ total vigorous MET. Analyses were performed with and without outlier adjustments with no differences in the results.

Short form McGill questionnaire and FIQR did not differ across sessions (*P* > 0.05) for both groups. Compared with healthy controls, individuals with FMS had significantly higher SF-MPQ (*P* < 0.001). Body composition and physical activity were not significantly different between groups (*P* > 0.05) with the exception of visceral adipose tissue (*P* < 0.05). Based on their BMI and IPAQ scores, participants on average were considered overweight or obese with moderate to high levels of physical activity. Although physical activity was not different between the groups, there was a wider range (higher SD) in participants with FMS especially for vigorous IPAQ scores (Table 1).

### 3.2. Baseline conditioned pain modulation

The analysis of PPTs for baseline CPM demonstrated no interaction between site, time, session, or group (*P* > 0.05). There was a main effect of site (F(1,40) = 76.187, *P* < 0.001, $\eta_{\text{p}}^{2}$ = 0.656), time (F(1,40) = 38.819, *P* < 0.001, $\eta_{\text{p}}^{2}$ = 0.493), and group (F(1,40) = 6.879, *P* = 0.012, $\eta_{\text{p}}^{2}$ = 0.147). Post hoc analyses showed there was an increase in PPTs at the deltoid and quadriceps muscles while the foot was submerged in the ice water bath and immediately after removal of the foot from the ice water bath, which signifies CPM (*P* < 0.005). These effects were significant for both groups; however, individuals with FMS had significantly lower PPTs than healthy controls at all time points (*P* < 0.01). In addition, PPTs were higher at the quadriceps muscle compared with the deltoid muscle (*P* < 0.01). When comparing the magnitude of CPM at baseline, there were no differences between sessions, sites, or groups (*P* > 0.05).

### 3.3. Pressure pain thresholds after washout period

When we examined whether PPTs returned to baseline after the 45-minute quiet rest or washout period, the results demonstrated no interaction between site, session, trial, or group (*P* > 0.05). There was a main effect of PPT trial (pre-exercise and postexercise or quiet rest) (F(1,40) = 6.243, *P* = 0.017, $\eta_{\text{p}}^{2}$ = 0.135), site (F(1,40) = 75.053, *P* < 0.001, $\eta_{\text{p}}^{2}$ = 0.652), and group (F(1,40) = 5.905, *P* = 0.020, $\eta_{\text{p}}^{2}$ = 0.129). Post hoc analyses showed that PPTs did not completely return to baseline after the 45-minute washout period and before the exercise or quiet rest (Table 2).

**Table 2:** Participants' mean ± SD PPT values, percent change after exercise and quiet rest sessions, and CPM.

	Site	Baseline PPT (before first CPM)	CPM first trial	PPT before exercise or quiet rest (after washout period)	PPT after exercise or quiet rest	Average percent change* (EIH)	Baseline PPT (before second CPM)	CPM second trial
Exercise session								
Healthy control	Deltoid	324.6 ± 120.7	21.1 ± 22.0%	351.1 ± 128.6	397.8 ± 147.3	13.7 ± 19.1%	360.9 ± 169.1	18.9 ± 24.0%
	Quadriceps	462.4 ± 162.1	13.1 ± 22.2%	467.8 ± 166.4	559.6 ± 210.7	18.5 ± 19.1%	532.6 ± 180.4	3.4 ± 17.2%
								
FMS	Deltoid	260.6 ± 122.6	16.6 ± 31.7%	264.5 ± 110.9	280.3 ± 126.3	7.0 ± 20.0%	246.6 ± 110.0	11.6 ± 19.9%
	Quadriceps	346.5 ± 144.3	12.8 ± 22.7%	359.7 ± 169.7	415.1 ± 203.5	18.1 ± 28.7%	367.3 ± 185.4	10.1 ± 26.6%
								
Quiet rest session								
Healthy control	Deltoid	308.9 ± 102.3	21.0 ± 17.4%	345.5 ± 94.0	323.9 ± 114.9	−5.7 ± 23.3%	342.6 ± 122.5	10.9 ± 12.7%
	Quadriceps	455.1 ± 169.1	16.8 ± 18.7%	485.0 ± 172.9	478.6 ± 168.7	−1.3 ± 19.2%	473.9 ± 153.0	9.4 ± 14.9%
								
FMS	Deltoid	247.5 ± 97.5	18.4 ± 26.9%	267.1 ± 117.4	249.2 ± 116.9	−5.3 ± 21.5%	260.0 ± 120.2	4.8 ± 18.0%
	Quadriceps	358.4 ± 172.3	9.3 ± 21.9%	374.4 ± 173.6	374.1 ± 177.3	0.1 ± 20.8%	379.7 ± 180.1	1.7 ± 22.1%

CPM, conditioned pain modulation; FMS, fibromyalgia syndrome; PPT, pressure pain threshold.

* Calculated as ([PPT after exercise or quiet rest − PPT before exercise or quiet rest (after washout period)]/PPT after exercise or quiet rest) × 100.

### 3.4. Baseline conditioned pain modulation responders and nonresponders

The SEM at the deltoid was 41.41 kPa for healthy control participants and 55.06 kPa for individuals with FMS. This corresponds to 13.37% change in PPTs for healthy controls (exercise session: 12 responders and 9 nonresponders; quiet rest session: 14 responders and 8 nonresponders) and 21.59% change in PPTs for individuals with FMS (exercise session: 9 responders and 12 nonresponders; quiet rest session: 8 responders and 13 nonresponders). The SEM at the quadriceps was 79.29 kPa for healthy control participants and 59.6 kPa for individuals with FMS. This corresponds to 17.55% change in PPTs for healthy controls (exercise session: 8 responders and 13 nonresponders; quiet rest session: 7 responders and 15 nonresponders) and 16.55% change in PPTs for individuals with FMS (exercise session: 8 responders and 13 nonresponders; quiet rest session: 7 responders and 14 nonresponders). Therefore, CPM responders and nonresponders varied by session and site.

### 3.5. Pressure pain thresholds after exercise and quiet rest

For PPTs, there was only a session × PPT trial (F(1,40) = 20.845, *P* < 0.001, $\eta_{\text{p}}^{2}$ = 0.343) interaction. Post hoc analyses showed that PPTs increased in the exercise session (EIH; *P* < 0.01) without any significant changes after quiet rest (*P* > 0.05) (Table 2). The absence of an interaction with site indicates local and systemic EIH. Similarly, the absence of an interaction with group indicates similar change in PPTs for the FMS and control groups; however, a main effect of group was significant (F(1,40) = 6.327, *P* = 0.016, $\eta_{\text{p}}^{2}$ = 0.137). Pressure pain thresholds were overall higher for healthy controls compared with people with FMS (*P* = 0.016). Thus, people with FMS had lower PPT than people without FMS, and both groups reported similar local and systemic EIH.

### 3.6. Conditioned pain modulation after exercise and quiet rest

**Figure 2.**
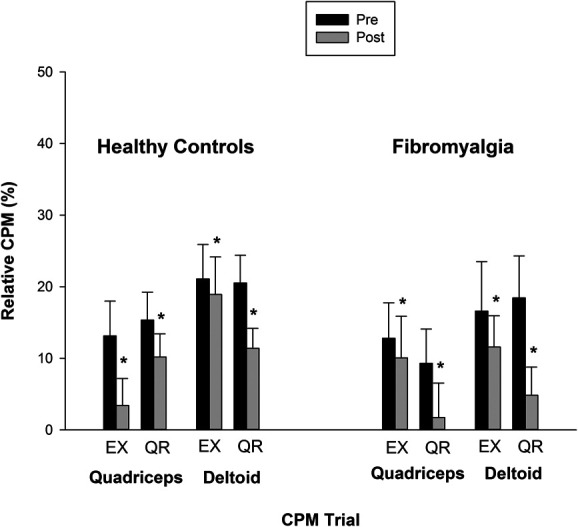
Influence of exercise on CPM. CPM decreased after exercise and quiet rest at the quadriceps and deltoid muscles for healthy control and participants with fibromyalgia. Significantly different compared with before exercise or before quiet rest (*). Data are presented as mean ± SE. CPM, conditioned pain modulation; EX, exercise; QR, quiet rest.

After exercise and quiet rest, there was no interaction between session, CPM trial, site, or group (*P* > 0.05), only a main effect of CPM trial (F(1,40) = 11.039, *P* = 0.002, $\eta_{\text{p}}^{2}$ = 0.216) and a main effect of site (F(1,40) = 6.732, *P* = 0.013, $\eta_{\text{p}}^{2}$ = 0.144) (Fig. 2). Post hoc analyses showed that CPM decreased after exercise and quiet rest (*P* = 0.002), and CPM was higher for the deltoid muscle compared with the quadriceps muscle (*P* = 0.013). Thus, CPM decreased after exercise and quiet rest at both sites.

### 3.7. Conditioned pain modulation responders and nonresponders after exercise and quiet rest

**Figure 3.**
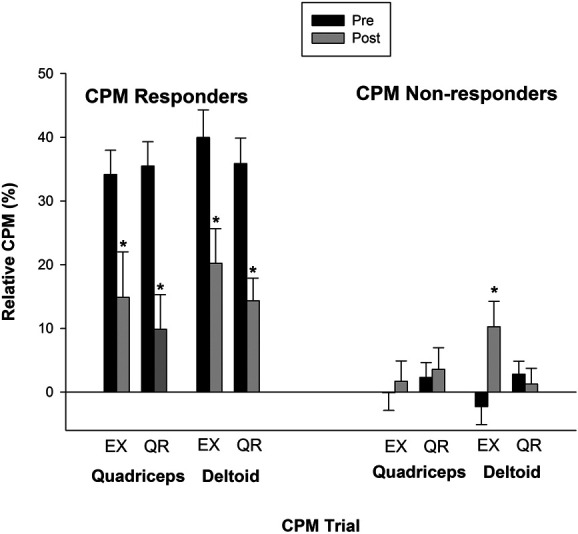
Influence of baseline CPM response on CPM response after exercise. For CPM responders, CPM decreased after exercise and quiet rest at the quadriceps and deltoid muscles for healthy control and participants with fibromyalgia. For CPM nonresponders, CPM increased after exercise only at the deltoid muscle with no significant change at the quadriceps muscle or after quiet rest. Significantly different compared with before exercise or before quiet rest (*). Data are presented as mean ± SE. CPM, conditioned pain modulation; EX, exercise; QR, quiet rest.

When evaluating CPM after exercise and quiet rest for CPM responders and nonresponders, there was an interaction between CPM trial with only CPM response in both sessions at both sites. There was no interaction with group (*P* > 0.05). The CPM response after exercise at the deltoid (CPM trial × CPM response: F(1,38) = 12.546, *P* = 0.001, $\eta_{\text{p}}^{2}$ = 0.248) and quadriceps muscles (F(1,38) = 7.552, *P* = 0.009, $\eta_{\text{p}}^{2}$ = 0.166) for CPM responders was reduced (*P* < 0.05), whereas for nonresponders CPM increased at the deltoid muscle only (*P* = 0.01; Fig. 3). Exercise restored CPM when measured distally from the exercising muscle in individuals with impaired CPM, and CPM was reduced for individuals with functional CPM at both sites after exercise. Baseline characteristics of responders and nonresponders at the deltoid site in the exercise session were not significantly different except for age (responders tended to be younger; 44.4 vs 54.9 years; *P* = 0.016) and total fat percent (responders tended to have more fat percent; 42.6 vs 36.9%; *P* = 0.036).

After quiet rest, CPM responders and nonresponders also behaved differently at the deltoid (CPM trial × CPM response: F(1,39) = 9.609, *P* = 0.004, $\eta_{\text{p}}^{2}$ = 0.198) and the quadriceps muscles (F(1,39) = 16.912, *P* < 0.001, $\eta_{\text{p}}^{2}$ = 0.302). Post hoc testing showed that for CPM responders, CPM was reduced after quiet rest for both muscles (*P* < 0.01) with no significant change for nonresponders (*P* > 0.05).

## 4. Discussion

The novel finding of this study was that a sustained isometric contraction restored CPM in individuals with attenuated CPM irrespective of health status (healthy or FMS). In contrast to the individuals with low-functioning baseline CPM, individuals who had a “normal” CPM before exercise had a decrease in CPM after exercise and quiet rest. These findings were consistent for people with and without FMS and may be attributed to the comparable age, sex, body fat percentage, and physical activity levels between the groups.

### 4.1. Conditioned pain modulation

Despite previous research demonstrating that people with chronic pain have impaired CPM,^28^ CPM was similar between individuals with FMS and healthy control participants; although others have shown variability in the CPM response for people with FMS with some individuals having functional CPM.^2^ One explanation is that individuals with FMS who participated in this study were higher functioning compared with participants in previous studies, as shown by the lower FIQR scores (38.6 vs 52.4–60.4 in past studies).^30,42^ Furthermore, our participants showed moderate to high levels of physical activity which have been previously linked to better CPM in young and older adults.^32,33^ Considering this study was advertised as the “exercise and pain study,” it is conceivable that individuals with FMS who contacted us were interested in exercise as an intervention and had higher levels of physical activity. Alternatively, the participants without FMS may be at risk of developing chronic pain because of their sex, age, and weight status (overweight)^7,13–15^ that likely contributes to having similar CPM as individuals with chronic pain.

Despite the presence of CPM both during and after the conditioning stimulus, the 50-minute washout period in this study was not sufficient; PPTs did not return to baseline, and CPM magnitude was significantly reduced after quiet rest. Piloting of this same protocol in young healthy adults resulted in similar CPM responses before and after 45 minutes of quiet rest. Future research should consider a longer washout period when using a CPM protocol in people who are primarily middle-aged overweight or obese women.

### 4.2. Conditioned pain modulation responders and nonresponders

Individuals who were classified as CPM nonresponders before exercise experienced an enhanced CPM after exercise systemically at the nonexercising muscle (ie, the deltoid), suggesting activation of descending inhibitory pathways. Whereas individuals who were classified as CPM responders before exercise, experienced a reduction in CPM after both exercise and quiet rest. Previously, we have shown CPM was reduced after exercise in young healthy adults who reported systemic EIH.^1^ Our results contribute to this body of literature showing that exercise may restore CPM in those with impaired CPM and parallels other studies showing similar CPM benefits using TENS and joint mobilizations. In addition, in individuals with neuropathic pain, duloxetine (serotonin–norepinephrine reuptake inhibitor) and tapentadol (opioid agonist and norepinephrine reuptake inhibitor) were effective in individuals with impaired CPM.^34,46^ The common neurotransmitter in both studies was norepinephrine, which is known to be activated with exercise.^4,35^

The lack of change in CPM at the exercising muscle in CPM nonresponders could be explained by the inhibitory effects of CPM which omits the spinal segment of the corresponding conditioning stimulus.^25^ Participants reported severe pain with our exercise protocol; thus, the exercise may have acted as a conditioning stimulus in which systemic inhibition may have omitted the quadriceps region; accordingly, no change was observed in CPM at the quadriceps. Another explanation is that the mechanism by which local hypoalgesia occurs after exercise is different than mechanisms for systemic hypoalgesia.

### 4.3. Exercise-induced hypoalgesia

The fatiguing component of the sustained submaximal isometric contraction is important for health benefits, such as neuromuscular adaptions that are integral for improvements in physical performance and quality of life.^12^ All participants met the task failure criteria and reported minimal lingering pain (2–3 minutes after exercise cessation). Furthermore, both groups reported EIH (ie, increase in PPTs) locally at the exercising muscle and systemically. Previous research has shown that individuals with FMS have either worse or more variable pain responses to exercise compared with healthy controls.^19,37^ The significant hypoalgesic response in individuals with FMS found in this study could be explained by the permission of medications use or the higher functioning group recruited in this study. In addition, the exercise task was individualized to the participants' strength (ie, 30% MVC). Others^19^ have used a set weight which could result in a higher intensity for people with FMS because of differences in baseline strength, resulting in different pain responses after exercise. In summary, the similar EIH response in both groups supports the use of submaximal isometric contractions as a pain-relieving tool in healthy and clinical populations.

### 4.4. Study limitations

Limitations include the use of medication and the sample size for the CPM response subgroups. Medication use was allowed to better mimic clinical settings where patients are often evaluated and treated while using these medications and the ethical reasons of asking participants to withhold medications for several days or weeks to participate in this study. Second, the sample size for the subgroups is relatively small resulting in a smaller effect size. Nevertheless, the results were similar using all CPM calculation methods, and therefore, a larger sample may not result in a significant change in the conclusions of this study.

## 5. Conclusion

This study was unique in that participants with FMS had similar body composition and physical activity levels as healthy control participants, who were primarily middle-aged and overweight or obese women. Both groups reported local and system EIH after isometric exercise held until task failure. In persons with low CPM, irrespective of health status, isometric exercise enhanced CPM at a site distal from the exercising muscle. Our results support the use of isometric exercise when initiating an exercise program especially for individuals with impaired CPM.

## Disclosures

The authors have no conflict of interest to declare.

This research was partially supported by NICHD of the NIH under award number R15HD090265 (M.H.B.), Marquette University Department of Physical Therapy, and the Clinical & Translational Rehabilitation Health Sciences Program.

## Appendix A. Supplemental digital content

Supplemental digital content associated with this article can be found online at http://links.lww.com/PR9/A153.

## Supplementary Material

SUPPLEMENTARY MATERIAL
