# Supplementary material for: Impaired conditioned pain modulation was restored after a single exercise session in individuals with and without fibromyalgia
Source: Pain Rep. 2022 Apr 1;7(3):e996. doi: 10.1097/PR9.0000000000000996 (PMC8984585; doi:10.1097/PR9.0000000000000996)
Supplement: SUPPLEMENTARY MATERIAL [file painreports-7-e996-s001.pdf]

IMPAIRED CONDITIONED PAIN MODULATION WAS RESTORED AFTER A SINGLE EXERCISE SESSION IN INDIVIDUALS WITH AND WITHOUT FIBROMYALGIA.

Ali Alsouhibani [1,2]
Marie Hoeger Bement[1]

1 Clinical & Translational Rehabilitation Health Science Program, Department of Physical Therapy, College of Health Sciences, Marquette University, Milwaukee, WI

2 Department of Physical Therapy, College of Medical Rehabilitation, Qassim University, Buraydah, Saudi Arabia

Corresponding Author: Marie Hoeger Bement

Department of Physical Therapy, Marquette University, 561 N 15 St, Milwaukee WI 53233, USA

Phone: 414-288-6738 Fax: 414-288-5987

Email: mariehoeger.bement@marquette.edu

Table SDC1. Description of self-reported questionnaires.

Questionnaire	Description
PARQ[4]	This questionnaire is a screening tool for physical activity readiness recommended by the American College of Sports Medicine (ACSM). All participants completed at the start of the first session.
SF-MPQ[3]	This questionnaire measures multiple aspects of current pain (affective, sensory, and cognitive). Higher scores represent greater pain. All participants completed at the beginning of each session.
FIQR[1]	This questionnaire evaluates mood and symptoms related to fibromyalgia and other components of health status during the past week. There are 3 domains: overall impact, symptoms, and function. Higher scores represent more severe symptoms. Participants with FMS completed at the beginning of each session.
ACR Diagnostic Criteria for Fibromyalgia (2010)[5]	The 2010 diagnostic criteria is a self-reported questionnaire that contains two subscales: Widespread Pain Index (WPI) across 19 body sites and Symptom Severity (SS). Individuals meet the criteria for fibromyalgia if they had a WPI of 7 or more and an SS score of 5 or more OR a WPI between 3 and 6 and an SS score of greater than or equal to 9 for at least 3 months. Participants with FMS completed at the beginning of the first session.
IPAQ[2]	This questionnaire is a self-reported measure of physical activity and sitting time in the past week. There are 4 domains: occupation, transportation, household, and leisure. All participants completed during quiet rest of the third session.

PARQ, Physical Activity Readiness Questionnaire; SF-MPQ, Short form McGill Pain Questionnaire; FIQR, Revised Fibromyalgia Impact Questionnaire; ACR, American College of Rheumatology; IPAQ, International Physical Activity Questionnaire

References

[1] Bennett R. The Fibromyalgia Impact Questionnaire (FIQ): a review of its development, current version, operating characteristics and uses. Clin Exp Rheumatol 2005;23:154.

[2] Craig CL, Marshall AL, Sjostrom M, Bauman AE, Booth ML, Ainsworth BE, Pratt M, Ekelund U, Yngve A, Sallis JF, Oja P. International physical activity questionnaire: 12-country reliability and validity. Med Sci Sports Exerc 2003;35:1381-1395.

[3] Melzack R. The short-form McGill Pain Questionnaire. Pain 1987;30:191-197.

[4] Sanders M, American College of Sports Medicine. ACSM's health/fitness facility standards and guidelines. : Human Kinetics, 2018.

[5] Wolfe F, Clauw DJ, Fitzcharles MA, Goldenberg DL, Katz RS, Mease P, Russell AS, Russell IJ, Winfield JB, Yunus MB. The American College of Rheumatology preliminary diagnostic criteria for fibromyalgia and measurement of symptom severity. Arthritis Care Res (Hoboken) 2010;62:600-610.
